# Interaction between adverse childhood experiences and polygenic risk in patients with bipolar disorder

**DOI:** 10.1038/s41398-020-01010-1

**Published:** 2020-09-22

**Authors:** Young-Min Park, Tatyana Shekhtman, John R. Kelsoe

**Affiliations:** 1 Department of Psychiatry, Ilsan Paik Hospital, Inje University, Goyang, Republic of Korea; 2 Department of Psychiatry, University of California San Diego, La Jolla, CA USA

**Keywords:** Clinical genetics, Molecular neuroscience

## Abstract

The interaction between genes and environment often occurs when they depend on one another. We hypothesized that adverse childhood experiences (ACEs) would interact with genetic predispositions to bipolar disorder (BD), demonstrating earlier age at onset (AAO) and worse clinical outcomes. We aimed to clarify the effects of the interaction between ACEs and genetic susceptibility using polygenic risk score (PRS) on AAO and clinical outcomes. Single nucleotide polymorphisms and clinical data, including ACEs, were obtained from the Bipolar Genomic Study, which contains a large sample of BD participants. A total of 1615 subjects with BD I were obtained and divided into two groups according to the presence or absence of ACEs and an additional four groups based on the number of ACEs (none versus one versus two versus ≥ three types). ACEs was evaluated using the childhood life events scale (CLES). BD–PRS was obtained from the Psychiatric Genomics Consortium, which compared BD patients and healthy controls. The BD–PRS was higher in the group with ACEs than without ACEs at most *p*-value thresholds. In multivariate linear regression analyses, both groups with more ACEs and higher BD–PRS were independently and interactively associated with an earlier AAO of BD; however, only greater ACEs were associated with worsened clinical outcome. These findings highlight the clinical importance of evaluating ACEs and polygenic risk in research of the etiology of BD.

## Introduction

The interaction between genes and environment (G × E) often occurs when they depend on one another^1^. For example, adverse childhood experiences (ACEs) are well-known environmental factors in the development of bipolar disorder (BD) in vulnerable individuals^2^. ACEs are potentially childhood traumatic events that can have negative, lasting effects on health and well-being, ranging from a parent loss or parental divorce to physical or emotional abuse^3^. Additionally, ACEs is thought to interact with vulnerable genes to induce the clinical development of symptoms of more severe BD, such as earlier age at onset (AAO)^1^.

An effect of ACEs on the AAO of BD was found in specific carriers of the SCL6A4 promoter genotype^4^. In addition, the negative effects of childhood sexual abuse on the AAO of BD may be amplified in carriers of the TLR2 rs3804099 risk genotype^5^; however, approximately 95% of positive findings from candidate gene studies have been estimated to be false positives^6^. Moreover, molecular data have confirmed that schizophrenia and bipolar disorders are highly polygenic^7^.

Researchers have begun to investigate polygenic effect using polygenic risk scores (PRSs)^8^. These scores were calculated after conducting a genome-wide association study (GWAS) in a discovery sample and analyzing single nucleotide polymorphisms (SNPs) for multiple *p*-value thresholds to predict BD in an independent target sample^9^. Each of these large numbers of SNPs in the target sample is weighted by their effect size in a discovery sample (GWAS) to reflect the polygenic effect of BD. Thus, high PRS could mean high BD genetic predisposition. Some investigators have found that individuals with high PRS instead of single SNP, and exposure to childhood abuse are particularly at risk for developing major depressive disorder compared to normal controls and rapid cycling within BD patients^10,11^; however, no previous studies have investigated the relationship between PRS and various ACEs as well as abuse. We hypothesized that ACEs interact with the genetic predisposition to BD, and that both of these factors would induce earlier AAO and worse clinical outcomes of BD. We also hypothesized that the group with more ACEs would demonstrate lower PRS due to G × E within BD patients.

The purpose of the present study was to clarify the effects of the interaction between ACEs and PRS on AAO and clinical outcomes.

## Participants and methods

### Participants

SNPs and clinical data, including ACEs, were obtained from the Bipolar Genomic Study (BiGS) by Bipolar Disorder Genetic Association Information Network and Translational Genomic Institute which is a large sample that was collected by the National Institute of Mental Health Genetics Initiative for Bipolar Disorder for large-scale GWAS in the United States^12^. These data were accessed by Dr. Kelsoe who is a member of the BiGS. All subjects provided written informed consent in accordance to protocols from local institutional review boards. From this cohort, we obtained 1 615 subjects with BD I, who were of European ancestry to heighten homogeneity. Participants provided written informed consent according to institutional review board protocols and were evaluated using the Diagnostic Interview for Genetic Studies (DIGS)^11,13^. DIGS is a specific clinical interview for evaluating mood and psychotic disorders through a semistructured design corresponding to a wide spectrum of DSM-IV criteria. The test–retest reliability (interval 4–10 days in the cross-site phase) was found to be high. Excellent inter-rater reliability was found for schizophrenia, bipolar disorder, major depression, and unipolar schizoaffective disorder. The DIGS’ scoring system includes the following clinical information: AAO, psychotic symptom severity (scored as 1 [fleeting], 2 [one episode], 3 [≥2 episodes], 4 [all episodes], or 5 [chronically psychotic]), history of mixed symptoms or episodes (scored as 0 [never], 1 [overlapping or mixed symptoms], or 2 [definite with at least one clear mixed episode according to the diagnostic and statistical manual of mental disorders, fourth edition]), suicidality assessment (scored as 0 [never], 1 [only passive death wishes], 2 [thought about suicide but never acted on], 3 [acted on with ambivalence/minimal consequences], or 4 [acted on with intent to die/serious consequences]), psychosis history, general impact of illness on life functioning (scored as 0 [never ill], 1 [no loss of employment or marital status], 2 [loss of employment or marital status but not disabled], 3 [disabled but living independently], or 4 [disabled and not living independently]), and substance misuse history (scored as 0 [no substance misuse], 1 [no substance abuse/dependence], 2 [brief usage (<2 years or <25% of illness)], 3 [remitting/relapsing], or 4 [chronic usage (>75% of illness)]). SNPs and ACEs were evaluated from the final dataset from the cohort.

### Genotyping and quality control

A detailed description of the sample and genotyping procedures has been previously described^11^. Genotyping of the BiGS sample was conducted using the Affymetrix Genome-Wide Human SNP Array 6.0 (Santa Clara, CA, USA). Initially, it consisted of 2200 BD patients with ACE data and 1436 healthy controls with 703,012 SNPs. We included only BD I cases with European ancestry and excluded healthy controls in the present study. The new sample underwent an extensive quality control process to eliminate individuals with >10% missing data and SNPs with poor allele clustering, duplicate errors, minor allele frequencies (MAFs) <0.01, Hardy–Weinberg equilibrium <10^−6^, and nonEuropean ancestry as indicated with principal component analysis. Finally, we retained 1615 BD I participants with 632,739 SNPs.

### Childhood life events scale (CLES)

ACEs were evaluated using the CLES which is a nine-item scale assessing various ACEs, including physical abuse, between 3 and 12 years of age^2^. The CLES score corresponds to the number of accumulated traumatic events during that period, with a range from zero to nine events. The CLES contains the following items: death of a parent, death of a sister or brother, onset of a chronic illness (e.g., juvenile diabetes), long-term hospitalization (≥1 month), permanent injury or disability (e.g., loss of a limb), physical abuse (PA), receiving a death or injury threat, leaving home unexpectedly (e.g., foreclosure by the bank), and serious unexpected life changes (e.g., a parent losing a job).

### Polygenic risk score (PRSs)

The PRSs were created using the odds ratios from the Psychiatric Genomics Consortium (PGC)^14^ data, which compared GWAS BD patients and healthy controls. This was the first large collaborative GWAS in BD patients that used the multinational PGC BD Working Group was comprised of 7481 patients with BD and 9250 controls^14^. Participant PRSs were obtained following the method described by Purcell and colleagues using PLINK software^15^. From the meta-analysis, SNPs were selected that had an imputation INFO score < 0.9, MAF < 0.02, and low linkage disequilibrium to each other (*r*^2^ < 0.25 within 500 kb). The meta-analysis results of SNPs for up to seven *p*-value thresholds (*p* = 0.0001, 0.001, 0.01, 0.05, 0.1, 0.2, and 0.3) were chosen to calculate the PRS in our current sample; therefore, the numbers of SNPs included were 124, 737, 4060, 14,161, 24,096, 40,640, and 54,577, respectively. The PRS was standardized using a mean of 0 and a standard deviation of 1 to aide in interpretation. For our main analysis, we used the threshold for PRS calculation that was associated with the highest explained variance in previous studies (i.e., *p* = 0.01)^10,16^. In our present sample, the threshold of *p* = 0.01 had a case-control explained variance of Nagelkerke *r*^2^ = 0.04.

### Statistical analysis

The patients were divided into two groups based on the presence or absence of ACE and into four additional groups based on the number of ACEs (none versus one versus two versus ≥ three types). Since all of the continuous variables except AAO did not conform to a normal distribution, Mann–Whitney and Kruskal–Wallis tests, as well as *t*-tests and analyses of variance were applied. The chi-squared test was applied to categorical variables. In addition, multivariate linear regression and binary regression analyses were performed to evaluate whether the AAO and clinical variables were associated with CLES and PRS scores. All statistical analyses were conducted using SPSS (version 21; IBM, Armonk, NY, USA) and SALT (version 2.5; Istech Inc., Goyang, Republic of Korea).

## Results

The demographic and clinical characteristics of the two groups (i.e., with and without ACEs) are presented in Table 1. At least one ACEs had been experienced during childhood by 63.6% of all participants. The AAO were significantly earlier in subjects, who had experienced ACEs and there was an association between ACEs presence and worse clinical outcomes of BD; however, the number of manic and depressive episodes per year did not differ between groups.

**Table 1:** Demographic and clinical characteristics in the presence and absence of adverse childhood experiences.

Variables	Total (*N* = 1615)	Group without ACE (*N* = 588)	Group with ACE (*N* = 1027)	*p*
Age at assessment (years; mean ± SD)	44.00 ± 12.91	43.75 ± 14.20	44.14 ± 12.11	0.43^a^
Sex (male/female)	588/1027	248/340	340/687	<0.001^**b^
AAO (years, mean ± SD)	18.69 ± 9.44	20.69 ± 9.91	17.55 ± 8.97	<0.001^**c^
Number of manic episodes per year	0.52 ± 1.29	0.45 ± 0.81	0.55 ± 1.50	0.224^a^
Number of depressive episodes per year	0.81 ± 2.02	0.87 ± 2.55	0.78 ± 1.64	0.46^a^
History of psychotic episodes (%)	69.1	69.3	69.0	0.94^b^
History of suicide attempts (%)	50.3	41.9	55.1	<0.001^**b^
History of mixed features (%)	48.0	40.2	52.5	<0.001^**b^
Presence of substance misuse (%)	58.3	53.0	61.3	<0.01^*b^
Presence of decreased functioning (%)	84.6	79.8	87.4	<0.001^**b^
Number of ACE (CLES score)	1.35 ± 1.42	0	2.12 ± 1.24	NA

Data are mean ± SD values.

*SD* standard deviation, *ACE* adverse childhood experience, *AAO* age at onset, *CLES* childhood life events scale, *NA* nonavailable.

*p < 0.01, **p < 0.001.

^a^Mann–Whitney test.

^b^Goodness-of-fit test.

^c^Independent *t*-test.

**Fig. 1:**
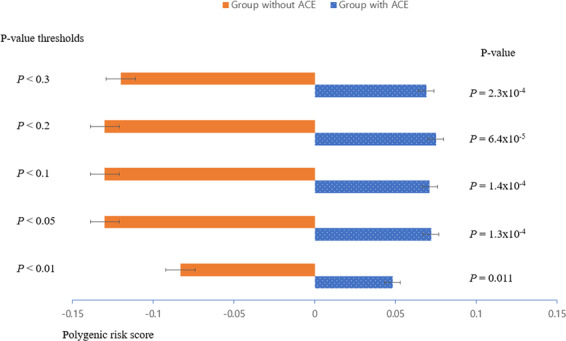
Polygenic risk scores for bipolar disorder. Comparison between groups with and without adverse childhood experiences in polygenic risk scores across five *p*-value thresholds.

**Fig. 2:**
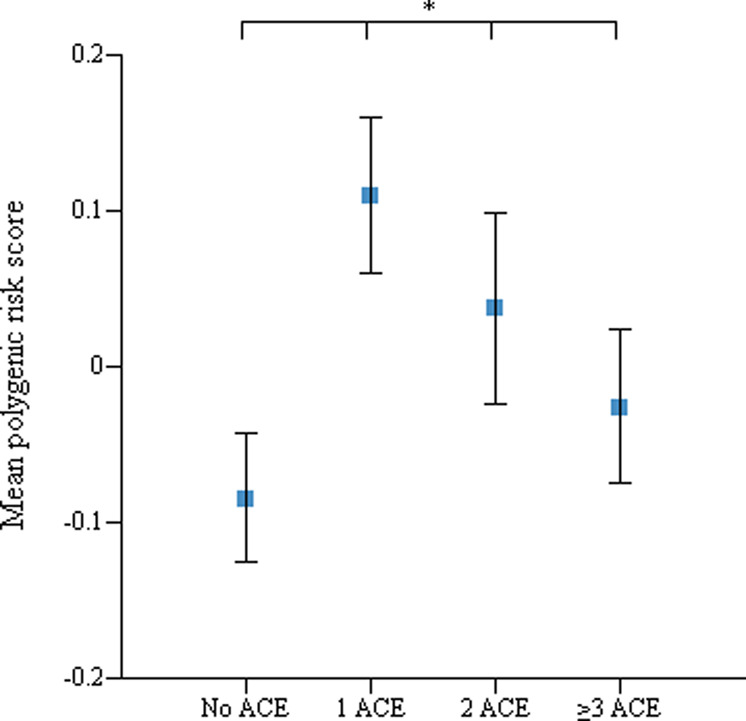
Polygenic risk scores and adverse childhood experiences. Mean polygenic risk scores according to groups based on the number of adverse childhood experiences (none versus one versus two versus ≥ three types) (*p=0.021).

BD–PRS was higher in the ACEs group than the group without ACEs for most thresholds of the *p*-value (*p* = 0.3, 0.2, 0.1, 0.05, and 0.01; Fig. 1) except two low *p*-value thresholds (*p* = 0.001 and 0.0001). Additionally, the mean PRS of the four groups based on the number of ACEs differed significantly among groups (df = 3, *p* = 0.021; Fig. 2). In multiple comparisons, there was a significant difference between the groups without ACEs and with one ACE (*p* = 0.015; Bonferroni corrected).

Multivariate linear regression analyses were performed to assess whether the number of ACEs and PRS were affecting the association with AAO. More ACEs and higher PRS were independently associated with an earlier AAO (Table 2); however, the number of major depressive and manic episodes was not associated with ACEs or PRS. Multivariate linear regression analyses were also performed to determine whether the four groups based on the number of ACEs and PRS influenced the association with AAO. Groups with more ACEs and higher PRS were independently and interactively associated with an earlier AAO (Table 3). Among other clinical outcomes, the presence of suicide attempts, worsening of functioning, and substance misuse were also associated with the number of ACEs (Table 4).

**Table 2:** Results of multiple linear regression analysis of age at onset and number of major depressive and manic episodes associated with adverse childhood experiences and polygenic risk score (continuous variables).

Variables	Coefficient	SE	*t*	*p*
*Age at onset*^a^				
Number of ACE (CLES score)	–1.21	0.15	–7.86	<0.001**
BD–PRS	–0.68	0.30	–2.30	0.022*
Interaction	0.29	0.16	1.79	0.074
*Number of manic episodes*^b^				
Number of ACE (CLES score)	0.047	0.084	0.57	0.57
BD–PRS	−0.026	0.052	−0.50	0.62
Interaction	0.066	0.112	0.59	0.55
*Number of depressive episodes*^b^				
Number of ACE (CLES score)	−0.15	0.13	−1.11	0.27
BD–PRS	−0.12	0.082	−1.47	0.14
Interaction	−0.083	0.17	−0.48	0.63

*CLES* childhood life events scale, *ACE* adverse childhood experience, *PRS* polygenic risk score, *BD* bipolar disorder.

*p < 0.05, **p < 0.001.

^a^Age at inclusion and sex were included as covariates in the analyses for age at onset.

^b^Age at inclusion, age at onset, and sex were included as covariates in the analyses for number of episodes.

**Table 3:** Results of multiple linear regression analysis for age at onset and number of major depressive and manic episodes between four groups based on the number of adverse childhood experiences (none versus one versus two versus ≥ three types) and polygenic risk score (continuous variables).

Variables	Coefficient	SE	*t*	*p*
*Age at onset*^a^				
Groups based on the number of ACE	–1.48	0.60	–2.47	0.014^*^
BD–PRS	–0.87	0.38	–2.32	0.02^*^
Interaction	−4.09	0.64	−6.35	<0.001^**^
*Number of manic episodes*^b^				
Groups based on the number of ACE	0.048	0.084	0.57	0.57
BD–PRS	−0.026	0.053	−0.50	0.62
Interaction	0.18	0.091	1.92	0.056
*Number of depressive episodes*^b^				
Groups based on the number of ACE	−0.15	0.13	−1.12	0.27
BD–PRS	−0.12	0.082	−1.48	0.14
Interaction	−0.018	0.14	−0.48	0.63

*CLES* childhood life events scale, *ACE* adverse childhood experience, *PRS* polygenic risk score, *BD* bipolar disorder.

*p < 0.05, **p < 0.001.

^a^Age at inclusion and sex were included as covariates in the analyses for age at onset.

^b^Age at inclusion, age at onset, and sex were included as covariates in the analyses for number of episodes.

**Table 4:** Results of the multiple binary regression analysis of clinical outcomes associated with adverse childhood experiences and polygenic risk score (categorical variables).

Variables	Coefficient	SE	df	*p*
*History of psychotic features*^a^				
Number of ACE (CLES score)	0.056	0.042	1	0.18
BD–PRS	−0.11	0.077	1	0.15
Interaction	0.067	0.04	1	0.098
*History of mixed symptoms*^a^				
Number of ACE (CLES score)	0.068	0.039	1	0.085
BD–PRS	−0.0008	0.074	1	0.91
Interaction	−0.006	0.041	1	0.89
*History of suicide attempts*^a^				
Number of ACE (CLES score)	0.18	0.039	1	<0001*
BD–PRS	−0.04	0.072	1	0.58
Interaction	−0.026	0.041	1	0.52
*Presence of worsening in life functioning*^a^				
Number of ACE (CLES score)	0.26	0.062	1	<0.001*
BD–PRS	−0.15	0.094	1	0.12
Interaction	0.055	0.065	1	0.40
*Presence of substance misuse*^a^				
Number of ACE (CLES score)	0.13	0.04	1	<0.001*
BD–PRS	0.027	0.073	1	0.72
Interaction	−0.033	0.042	1	0.43

*CLES* childhood life events scale, *ACE* adverse childhood experience, *PRS* polygenic risk score, *BD* bipolar disorder.

^a^Age at inclusion, age at onset, and sex were included as covariates in the analyses for all clinical outcomes.

^*^P < 0.001.

## Discussion

Ours is the study to use PRS and ACEs to examine the impact of the G × E interaction on the AAO and clinical outcomes of BD patients. We found that PRS was higher in the group with ACEs than without ACEs for most *p*-value thresholds and PRS, the four groups that were divided based on the number of ACEs, and their interaction induced earlier AAO.

Most previous studies have been limited by investigating only one or a few SNPs; however, candidate gene studies may miss some important genetic aspects of BD that is polygenic in nature. Recent studies have suggested that a high BD–PRS may be associated with the presence of psychotic features during mood episodes^17,18^, while BD–PRS has been suggested to not be associated with an earlier AAO^19^. An additional recent study reported the interaction between childhood maltreatment and PRS in patients with BD and found that BD–PRS and childhood maltreatment interacted to increase the risk of rapid cycling^11^.

In the present study, BD–PRS was higher in the group with ACEs than without ACE for most of the *p*-value thresholds (*p* = 0.3, 0.2, 0.1, 0.05, and 0.01; Fig. 1). This finding indicates that individuals with exposure to ACEs have a higher genetic predisposition toward BD. Thus, it can be hypothesized that the parental genetic susceptibility to BD may invoke ACEs on their offspring because of psychopathological factors such as high aggression, poor impulse control, and labile affect^20^. Otherwise, offspring with BD may bring parental maltreatment or ACEs upon themselves. These results suggest that ACEs may act as an environmental factor and a mediator connecting ACE and genetic susceptibility.

Etain et al. introduced the unresolved chicken-and-egg debate regarding the relationship between ACEs and BD in their review article^21^. Authors addressed the following four hypotheses about this relationship:

1. The parents or caregivers of patients with BD may demonstrate higher levels of expressed emotions, including critical, hostile, or overinvolved attitudes^22^, which may produce ACEs, such as emotional abuse^21^.
2. The childhood behavioral disturbances linked to early comorbid conduct or disruptive problems^23,24^ and attention deficits with hyperactivity^25^ may cause ACEs or abuse by parents or caregivers^21^.
3. Parental bipolar genetic loading and its psychopathology may lead to the disease in offspring and an elevation in the likelihood of ACEs^20^.
4. The intergenerational transmission of childhood trauma in BD families may support the interpretation of an association between childhood trauma and BD^21^.

Notably, our present findings support the first and third hypotheses.

Next, only BD–PRS did not correlate with AAO. This finding was consistent with a recent study with a larger sample, where BD–PRS was not found to be significantly associated with AAO; however, in multivariate linear regression analyses with PRS and the number of ACEs, both were independently associated with an earlier AAO in BD patients. In another multivariate linear regression analyses, four groups based on the number of ACEs and PRS were independently and interactively associated with an earlier AAO. Thus, it can be assumed that individuals with more BD-associated risk alleles and ACEs can be expected to cross the liability threshold earlier and have an earlier onset. Our findings were inconsistent with the only other study evaluating the interaction between BD–PRS and childhood maltreatment in 402 French and Norwegian patients with BD, which did not discover any relationship between BD–PRS and childhood maltreatment with AAO. We consider that since our participants consisted of only BD I patients with multiple recurrences, while only 74% of patients in the alternate study had BD I, this could reflect low BD–PRS. Another discrepancy is that we examined various childhood traumatic events, including childhood maltreatment, while they examined only childhood maltreatment when childhood trauma was evaluated; however, it is still difficult to draw conclusions due to the lack of research^26^.

Moreover, higher BD–PRS was not associated with worsened clinical outcomes. Contrarily, higher numbers of ACEs were associated with worse clinical outcomes, including suicide attempts, decreased functioning, and substance misuse, unlike BD–PRS. This appears to be due to the decreased influence of PRS than anticipated on clinical outcomes compared to ACEs. Current literature has revealed that the presence of only ACEs or childhood maltreatment are related to poor clinical outcomes^27^. Thus, the presence of ACEs appears to be more powerful predictor of poorer clinical outcomes than BD–PRS. However, both of PRS and ACE were not significantly related to the number of episodes of BD. According to two-hits model of susceptibility, childhood stressors interacts genetic factors, which lead to a susceptibility and then adulthood stressors may lead to recur and make episodes^28^. Thus, future studies are needed to investigate adulthood stressors as well as ACEs.

The present study was subject to some limitations. First, healthy controls were not included; therefore risk ratios between BD patients and healthy controls could not be determined. Second, recall bias associated with the retrospective study design may have been present ACEs recall may be affected by the mood of BD participants. Third, the PRS can only assess the level of SNPs, not the epigenetic level. Thus, future studies are needed to investigate the effects of epigenetics and polygenicity in BD. However, previous study found that BD patients with higher SPR–PRS had poor treatment response to lithium than those with lower SPR–PRS. Thus, BD–PRS do not necessarily mean the indicator of poor prognosis of BD. Forth, the present study just investigated the number of ACEs. Thus, future studies need to investigate not only the number but also the severity of ACEs using more integrative approaches. Notwithstanding these limitations, the present study has some significant strengths, such as the large sample size and using various data evaluations from patients with BD using structured questionnaires.

Both ACEs and BD–PRS were independently and interactively associated with an earlier AAO of BD. These findings highlight the clinical importance of evaluating ACEs and polygenic risk in understanding the etiology of BD.
